# *Staphylococcus epidermidis* is a safer surrogate of *Staphylococcus aureus* in testing bacterial filtration efficiency of face masks

**DOI:** 10.1038/s41598-023-49005-4

**Published:** 2023-12-09

**Authors:** Silvia Chiera, Francesca Bosco, Chiara Mollea, Amelia Piscitello, Rajandrea Sethi, Giandomenico Nollo, Iole Caola, Francesco Tessarolo

**Affiliations:** 1 Department of Industrial Engineering, University of Trento, Trento, Italy; 2 DISAT-Department of Applied Science and Technology, Politecnico di Torino, Torino, Italy; 3 DIATI-Department of Environment, Land and Infrastructure Engineering, Politecnico di Torino, Torino, Italy; 4 Microbiology and Virology Department, Azienda Provinciale per i Servizi Sanitari di Trento, Trento, Italy

**Keywords:** Biological techniques, Microbiology

## Abstract

Face masks play a role in reducing the spread of airborne pathogens, providing that they have a good filtration performance, are correctly fitted and maintained. Bacterial Filtration Efficiency (BFE) is a key indicator for evaluating filtration performance according to both European and US standards, requiring the use of *Staphylococcus aureus* loaded aerosol. However, the generation and handling of a Biohazard group 2 bacterium aerosol require a careful management of the biological risk and pose limitations to the accessibility to this method. To mitigate these drawbacks, we investigated the use of *S. epidermidis* ATCC 12228, a Biohazard group 1 bacterium, as surrogate in BFE test. To this end, tests with the surrogate strain were performed to tune the method. Then, three face mask models, representative for both surgical and community masks, were tested according to the standard method and then using an aerosolized suspension of *S. epidermidis*. BFE% values were calculated for each mask model and tested microorganisms. Results showed that BFE test can be performed using the *S. epidermidis* instead of *S. aureus*, preserving results validity and turnaround time, but reducing residual risk for laboratory operators.

## Introduction

Bacterial Filtration Efficiency (BFE) is a key indicator of the filtration performance of face masks according to several standard test methods, including EN 14683:2019^1^ in Europe and ASTM F2101-19^2^ in the United States. BFE testing methods specify the use of *Staphylococcus aureus* ATCC 6538 aerosol and a multistage impactor. However, the generation and handling of *S. aureus* aerosol, although in Biosafety Level 2 (BSL-2) laboratories, require a careful evaluation of the biological hazard risk, make difficult the environmental control measures, and limit the accessibility to this method. Indeed, according to the WHO Laboratory Biosafety Manual^3^, any procedure, that intentionally or accidentally produces liquid or solid particles, suspended in the air, is to be considered an aerosol-generating one.

In this context, the use of a less pathogenic, or preferably, non-pathogenic surrogate strain, for the generation of the loaded aerosol for BFE testing, could have a relevant impact in lowering the residual risk associated with the testing activity as well as widening the accessibility of this test to a larger number of laboratories and testing facilities. Moreover, at the end of the test, residual aerosol, microbial suspensions, and contaminated equipment or disposables can be decontaminated and discarded in a safer way.

Microbial surrogates play an important role as alternative biological indicators that can mimic survival and growth properties of related pathogens. Since the early days of microbiology, surrogate bacteria were used in place of a target pathogen for research activities, and their use has been essential part of pathogen studies because they are harmless microorganisms which have similar resistance properties to the pathogenic ones. Usually, surrogates are selected among well-known organisms that have specific characteristics and a long non-pathogenic history. In the review by Busta et al.^4^, the ideal surrogate is defined as a non-virulent strain that retains all the other characteristics of the pathogen (including susceptibility to antimicrobials or specific environmental conditions) except its pathogenicity.

*Staphylococcus epidermidis* ATCC 12228*,* a Gram-positive bacterium*,* ubiquitous and primarily harmless commensal of human skin and belonging to the human respiratory microbiota, has already been proposed as an effective surrogate of the pathogenic coagulase-positive *Staphylococcus aureus* both for clinical and food applications^5–8^. It has been previously used as surrogate to study an innovative water spa disinfection mechanism based on silver ions^7^ and in the greywater treatment based on an UV disinfection process^6^. *S. epidermidis* was also successfully used as surrogate test strain in antimicrobial tests performed on silver/plasma polymer nanocomposite coating on cotton fabric^8^.

The present study was aimed at investigating whether *S. epidermidis* ATCC 12228, a Biohazard group 1 microorganism^9,10^, could be exploited as a valid surrogate of *S. aureus* in the BFE test as per the standard EN 14683^1^. To this end, tests were performed to standardize the procedure with the selected surrogate strain, then bacterial filtration results, obtained using either *S. aureus* or *S. epidermidis*, were compared to validate the use of the surrogate microorganism for testing BFE of face masks.

## Results

### BFE test using *S. epidermidis*

In order to obtain statistically valid BFE test results, EN 14683:2019 specifies that positive controls should have a number of colony forming units (CFUs) ranging between 1.7 and 3.0 × 10^3^. The relationship between optical density (OD) values and CFU/mL was established using a 24-h old culture of the *S. epidermidis* strain. Different dilutions were prepared to identify the best concentration of the bacterial suspension to be nebulized.

**Figure 1:**
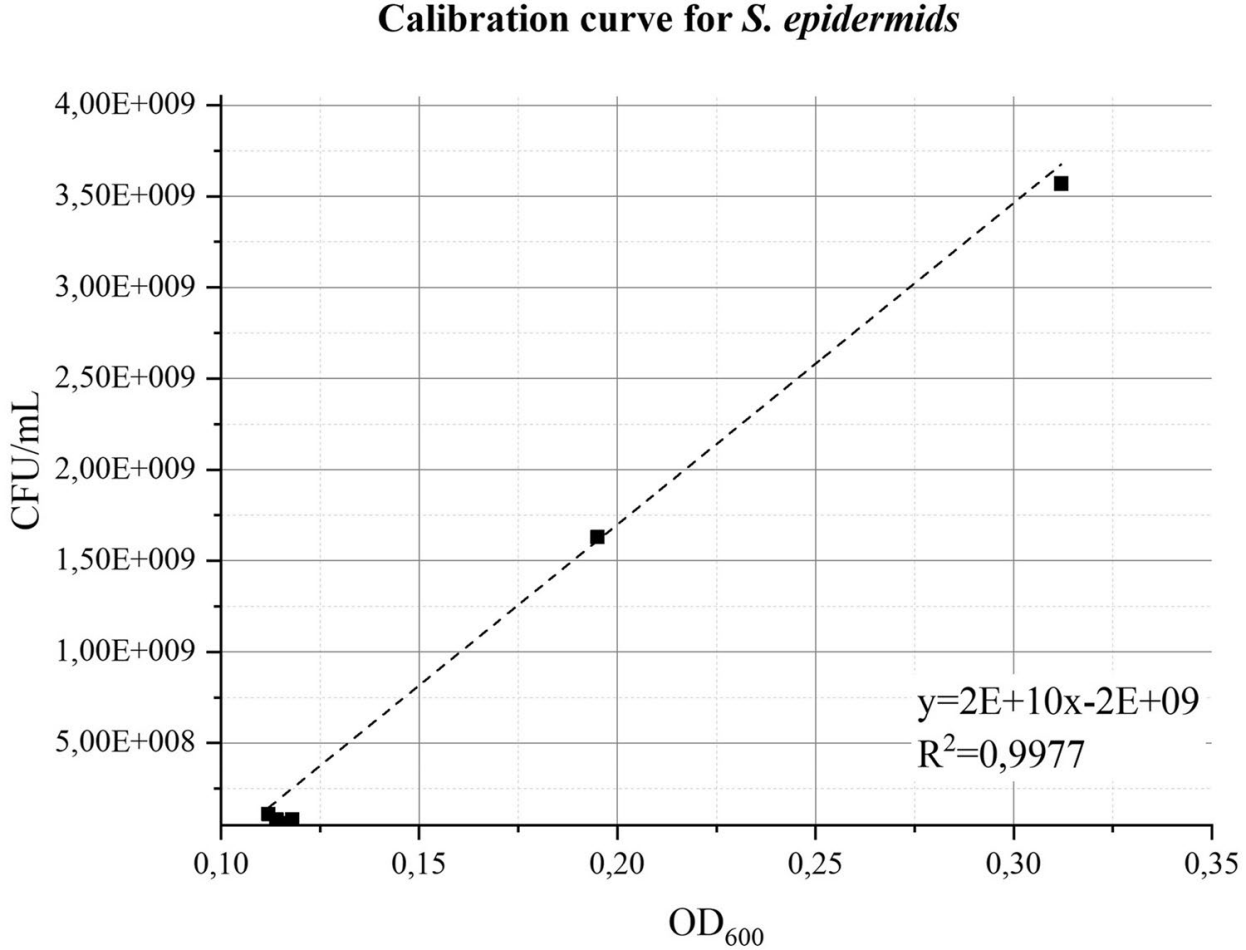
Calibration curve, correlating bacterial concentration (CFU/mL) to OD_600_ for a bacterial suspension obtained from the dilution in peptone water (PW) of a 24 h culture of *S. epidermidis* in Tryptone soy broth (TSB) at 37 °C.

Data indicating the bacterial concentration (CFU/mL) vs. optical density at 600 nm (OD_600_) are presented in Fig. 1, while the linear regression, in the OD_600_ range from 0.1 to 0.3, is reported in Eq. (1):

1 $$Bacterial \, concentration \left(\frac{CFU}{mL}\right)=2\times {10}^{10} O{D}_{600}-2\times {10}^{9}$$

A suspension of *S. epidermidis* with an OD_600_ in the range 0.112 ÷ 0.118 was prepared; the corresponding values of CFU/mL, from 0.6 × 10^8^ to 1.0 × 10^8^, were obtained applying Eq. (3). This bacterial suspension was further diluted with peptone water (PW) to obtain a final concentration of 1.6 × 10^4^ CFU/mL that allows to respect the microbial concentration indicated in the Norm. The suspension was nebulized at a flow rate of 0.7 mL/min using the test rig configuration presented in the Materials and Methods section. Results obtained in twelve positive controls confirmed that the suspension concentration ensures to obtain, in positive controls, a number of CFU always within the range specified by the standard. The average value of the CFU in positive control was 2.41 × 10^3^ ± 0.69 × 10^3^. The standard method also requires checking the aerosol size distribution. More specifically, the standard specifies that the Mean Particle Size (MPS) value should be in the range 2.7–3.3 µm. Under the above reported test conditions, repeated independent tests, 12 runs, showed an average value of the MPS equal to 2.71 ± 0.16. Results obtained from the analysis of the positive control runs, performed in different experimental sessions, are also summarized in Table 1 and presented Suppl. Fig. 1 of the supplementary materials.

**Table 1:** Aerosol particle size distribution obtained using *S. epidermidis*.

Stage	50% effective cut-off diameter (µm)	CFU
1	7.0	187 ± 67
2	4.7	198 ± 55
3*	3.3	607* ± 227
4*	2.1	579* ± 182
5*	1.1	824* ± 314
6*	0.65	12* ± 13
Total CFU		2.41 × 10^3^ ± 0.69 × 10^3^
MPS (µm)		2.71 ± 0.16

Mean values over twelve positive controls, performed in different experimental tests, are presented with their standard deviations. CFUs are listed together with total CFUs and MPS values.

*From 3rd to 6th impactor stages, CFUs have been enumerated by means of the “positive hole” correction.

Using the setting conditions identified above, BFE tests have been carried out on five equivalent samples of mask model A, type II R (see Table 4), to address repeatability of measurements.

The average number of CFU, at each impactor stage, is shown in Suppl. Fig. 2, distinguishing between data obtained from initial positive control (IPC) and final positive control (FPC). Results are summarized in Table 2 and presented in Suppl. Fig. 2, indicating the average of total CFU and MPS values obtained from the five replicated measurements on mask model A. Both parameters comply with standard specifications, being the total CFU of the IPC and the FPC respectively equal to 2.25 × 10^3^ and 2.76 × 10^3^ and the related MPS 2.75 and 2.70 µm.

**Table 2:** Dimensional distribution of the *S. epidermidis* aerosol expressed as mean of the total plate counts of the two positive controls.

Stage	50% effective cut-off diameter (µm)	IPC (CFU)	FPC (CFU)
1	7.0	179 ± 87	195 ± 32
2	4.7	206 ± 75	225 ± 60
3*	3.3	638* ± 237	789* ± 202
4*	2.1	518* ± 204	579* ± 144
5*	1.1	702* ± 318	958* ± 99
6*	0.65	9* ± 5	13* ± 6
Total CFU		2.25 × 10^3^ ± 900	2.76 × 10^3^ ± 500
MPS (µm)		2.75 ± 0.20	2.70 ± 0.1

Initial positive control (IPC) and final positive control (FPC) values are listed together with total CFU and MPS.

*From 3rd to 6th impactor stages, CFUs have been enumerated by means of the “positive hole” correction.

The BFE test, carried out on the model A face mask showed also good measurement repeatability with BFE values of the five replicates in the range 99.82–99.95%, an average value of 99.92%, and a repeatability standard deviation equal to 0.06%. Average BFE value was in line with the performance requirements for a type IIR medical face masks (≥ 98%). Overall, tuning test showed the technical feasibility of using *S. epidermidis* ATCC 12228 as a surrogate of *S. aureus* ATCC 6538 without major modification to the current BFE test method specified in the standard.

### Comparative BFE measurements using *S. epidermidis* and *S. aureus*

**Figure 2:**
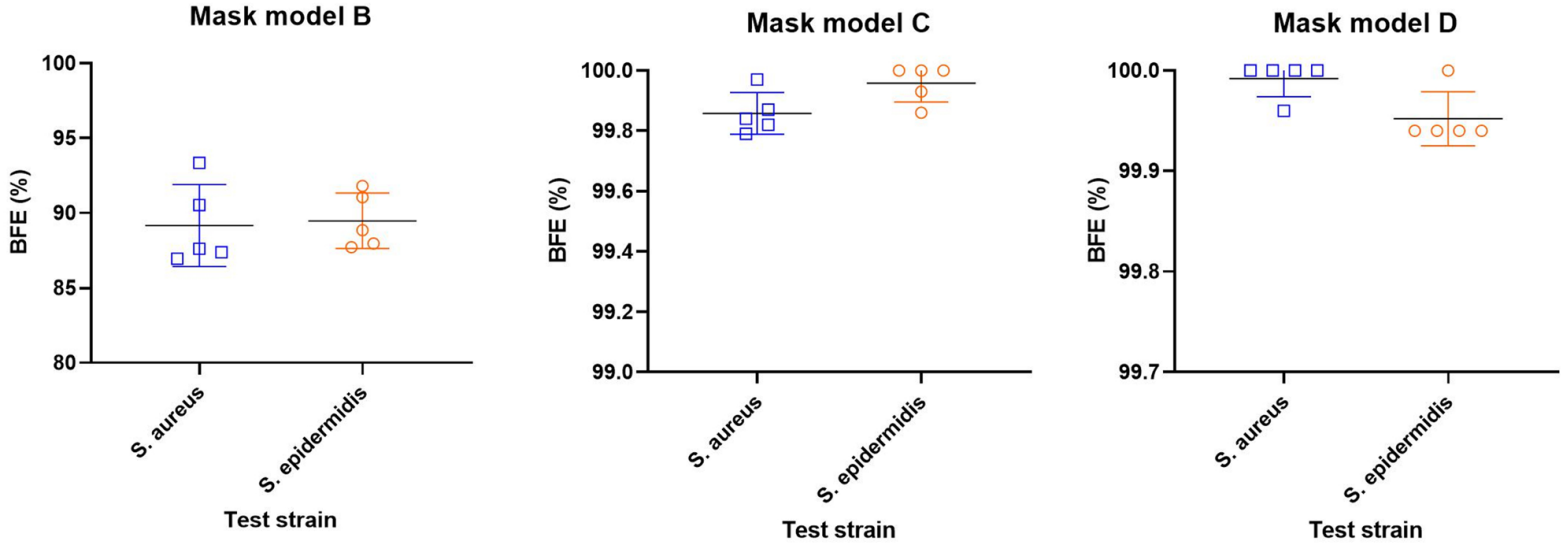
BFE (%) values collected from the three mask models (five replicated measurements each) using *S. aureus* and *S. epidermidis*.

To validate BFE test measurements performed using *S. epidermidis* as a surrogate of *S. aureus*, comparative tests were performed, and data were statistically analysed. BFE data obtained using either *S. aureus* or *S. epidermidis* aerosols are shown in Fig. 2 and summarized in Table 3. Differences between mean BFE values obtained using the two microbial strains on the same mask model are reported in the last column of Table 2, ranging from −0.04% to 0.32%, respectively for model D and B. ANOVA two-way test indicated no statistically significant difference between BFE data obtained with the two tested strains. On the other hand, ANOVA test indicated that both test methods were able to distinguish mask model B from C and D based on their BFE performance, confirming the ability to correctly discriminate face masks compliant with EN 14683 minimal requirements for surgical masks (i.e. > 95%) from other masks having lower BFE performance. Indeed, BFE values of the model B showed statistical difference from mask C and D, irrespectively of the microbial strain used for the test (p < 0.05).

**Table 3:** Results of the comparative BFE (%) test executed using *S. aureus* and *S. epidermidis*.

Mask model ID	BFE (%), mean ± SD		BFE difference, value (95% CI)
	*S. aureus*	*S. epidermidis*	
B	89.15 ± 2.74	89.47 ± 1.85	0.32 (−1.88 to 2.52)
C	99.86 ± 0.06	99.96 ± 0.06	0.1 (−2.10 to 2.30)
D	99.99 ± 0.02	99.95 ± 0.03	−0.04 (−2.24 to 2.16)

Differences of BFE values and their confidence intervals (CI) are also reported.

**Figure 3:**
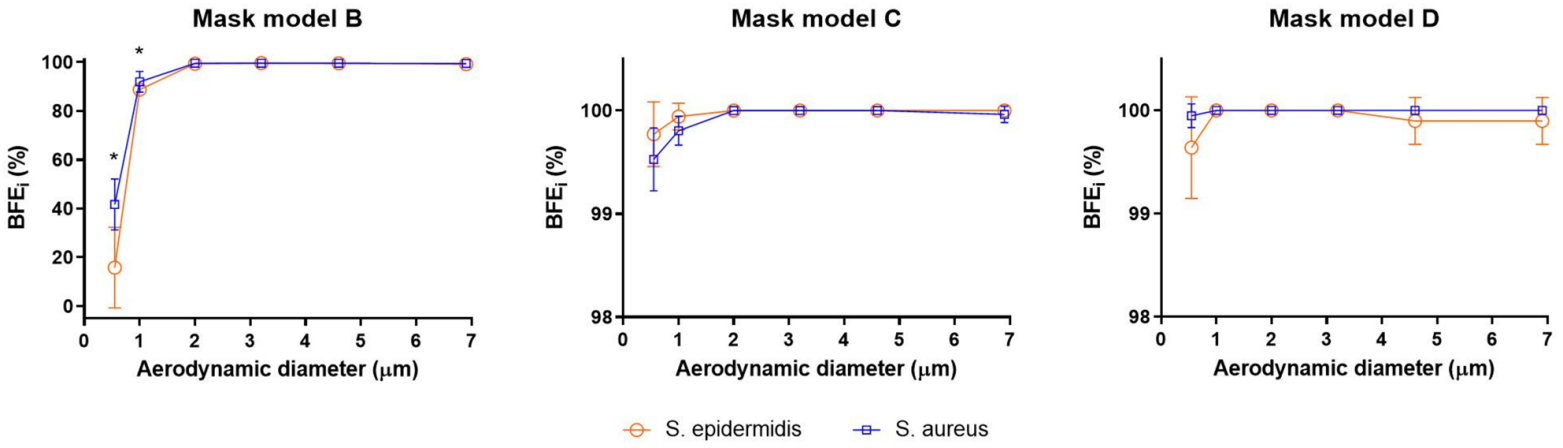
Spectral filtration performance (BFE_i_) for the two types of bacterial strains: *S. aureus* (blue) and *S. epidermidis* (orange). Data of each tested mask model (B, C, D) is presented in a separate graph. Experimental values N = 5, *p-value < 0.05.

Figure 3 summarizes filtration performance obtained using the two microbial strains as a function of the aerodynamic diameter of the aerosol particles. The comparison of spectral BFE (BFE_i_) data confirmed a substantial equivalence of the filtration results obtained using either *S. aureus* or *S. epidermidis*. ANOVA two-way test applied to spectral BFE data, showed no significant differences in filtration performance, except when small particles were filtered by mask model B. Only in this case, significantly lower BFE_i_ results were obtained for particles having 0,65 µm and 1.1 µm aerodynamic diameter when tested with *S. epidermidis*.

## Discussion

Covid-19 pandemic showed that the proper use of face masks can impact on the spreading of airborne pathogens and related diseases, and that a significant reduction of Sars-Cov-2 infective risk, varying from 17.4 to 3.1%, was associated to the use of a face mask^11^. The emergency due to Covid-19 pandemic triggered the production of new mask models and the investigation of alternative designs and materials for mask filters as a consequence of the capillary use of masks among workers and citizens^12^. New products, such as community masks, entered the market in a variety of different models. The demand for face masks increased as well as the need to test their safety and efficacy as recommended by WHO interim guidance^13,14^.

For decades, prior to the Covid-19 pandemic, only a small number of for-profit private laboratories globally conducted regulatory tests on masks for medical use^15,16^. On the contrary, after the starting of the pandemic, the demand of testing face masks performance increased as the natural consequence of a sudden market demand^12,17^. A range of organizational and logistic problems raised, not only at the production level, but also in testing face mask performance and safety^16^. This unprecedented demand for mask testing, including BFE one, was temporarily covered, in some countries, by both accredited and non-accredited laboratories^12,17^, stimulating a critical revision of the standard methods, by identifying weak methodological aspects and evidencing possible improvements^17,18^. The rational of the present study emerged in this context, with the aim to improve safety aspects and accessibility of BFE testing methodology.

The BFE testing methodology, specified in the current European^1^ and US standards^2^, requires the generation and handling of *S. aureus* aerosol, a Biohazard level 2 microorganism. WHO guidelines indicate that any procedure, that intentionally or accidentally creates liquid or solid particles suspended in the air, is considered an aerosol-generating one^3^. When a laboratory procedure requires the generation and use of aerosols, containing biological hazardous or pathogenic load, the definition and application of risk control measures are mandatory to minimize aerosol volume and/or diffusion. The current standard BFE methodology^1,2^, requiring the generation of aerosol containing the pathogenic strain *S. aureus*, limits the implementation of BFE testing activities to laboratories with adequate biosafety level (e.g. BSL-2 lab).

Inert particles, such as sodium chloride^19^ or polystyrene latex spheres^20^, were also widely investigated and used as substitute for biological particles in filtration tests^21,22^. However, different physical properties between inert particles and biological particles may result in different filtration efficiencies. Several particle features such as shape, dielectric property, and surface charge have a relevant impact on filtration efficiency and pose serious limitation in using common inert particles to replace biological particles to determine efficiencies of air filters^23^. Therefore, although aerosolization of human pathogens is generally discouraged^20^, its use is still part of the current standard BFE testing procedures^1,2^.

The use of a non-pathogenic surrogate microorganism for BFE test represents an opportunity to reduce safety issues and improve accessibility to BFE test to a larger number of laboratories, thus increasing the potential of generating a higher amount of BFE data and facilitating independent verification of claimed performance and quantitative product comparison.

*S. epidermidis* was selected as a surrogate for *S. aureus* in the BFE test, also considering its presence as a component of the respiratory microbiota. Given the absence of prior literature describing the use of *S. epidermidis* as a surrogate in the BFE test, a series of repeated experiments were conducted to optimize the standard method with *S. epidermidis* ATCC 12228 as the surrogate strain. This optimization allowed for the convenient determination of the suspension concentration prior to the BFE test, thereby reducing the variability of the results. Replicated tests were performed, involving viable cell counts both at the beginning and the end of each experiment, to ensure compliance with the standard specifications in terms of CFU and MPS of the positive controls. These results remained consistent across a range of air temperatures (20–27 °C) and relative humidity levels (40–55%). Complete BFE tests conducted using the surrogate microorganisms consistently produced reliable filtration efficiency values and demonstrated a high degree of repeatability. This was particularly important given the potential variations within equivalent products (same model, lot, and production date), as previously reported^24^.

To validate the suitability of *S. epidermidis* as a surrogate for *S. aureus* in BFE tests, various types of masks were tested within the same experimental setup, employing both bacterial strains. Statistically equivalent BFE results were obtained regardless of the tested strain. Furthermore, the results of spectral filtration efficiency (BFE_i_) showed a high degree of overlap for both strains.

*S. epidermidis* ATCC 12228 and *S. aureus* ATCC 6538 belong to the same *genus* and their size is comparable. Thompson et al. reported that also the sizes of aerosolized particles of *S. epidermidis*, and other staphylococci, are within the breathable range (1.1–2.1 µm) and thus suitable to be used in BFE tests^25^. According to our MPS results, both strains were effectively embedded in PW droplets and carried through the aerosol stream generated in the BFE test rig, resulting in aerosol size distributions with equivalent MPS. These results supported the effective use of the surrogate strain for obtaining equivalent filtration efficiency results.

Djeghdir et al. reported a strong correlation between bacterial and viral filtration efficiency when the pathogenic load was contained in droplets in the 2–3 µm range, indicating that BFE of face masks depends mainly on the size of the airborne droplet, rather than on the size of the infectious agent contained in that droplet^26^. Similarly, our results indicate that the BFE measurements obtained with both microorganisms were comparable both in terms of overall filtration efficiency and spectral filtration efficiency. Minor differences between the BFE spectral values detected in the community mask model B, resulted in a more conservative estimation of BFE when using the surrogate strain than the standard one.

Considering that mask filtration efficiency is a multifactorial process that depends on mask model and design, and on the type and number of filter layers, the obtained results on surgical mask category I, II and IIR, could be extended to other mask types, previously verifying the BFE equivalence of the surrogate strain^27^.

## Conclusions

The results obtained in the present work support the use of *S. epidermidis* ATCC 12228 as an effective and safer surrogate of *S. aureus* ATCC 6538 in testing BFE of face masks using the very same test rigs specified by the current standards. The test turnaround time remained unaffected, while the residual risk for laboratory operators and individuals involved in the decontamination of testing equipment was significantly reduced by eliminating the need for a BSL-2 lab. In addition, the accessibility to BFE testing methods is expanded allowing a higher number of laboratories and test facilities to perform tests using the surrogate *S. epidermidis* instead of the pathogenic *S. aureus*. In our opinion, the presented results could be considered for a revision of the current EN 14683:2019^1^, in Europe, and ASTM F2101-19^2^, in the United States, standards for testing BFE of surgical face masks.

## Materials and methods

### Bacterial Filtration Efficiency test according to standard EN 14683:2019

The test method and the test rig for BFE measurement of surgical masks is specified in Annex B of the European Standard EN 14683:2019 + AC^1^. The BFE test method requires the generation and control of a two-phase airflow which is forced to pass through the mask material and a multiple stage Andersen-type impactor^28,29^.

The standard method was reported elsewhere^24^ and is briefly described as follow. A bacterial suspension of *Staphylococcus aureus* ATCC 6538 is diluted in peptone water (PW) up to a concentration of 5 × 10^5^ CFU/ml to be aerosolized during the experimental session. The aerosolized suspension is obtained, using a nebulizer fed by a motorized syringe or a peristaltic pump with 0.01 ml/min of the bacterial suspension. The aerosol is injected in a vertical glass cylinder with a diameter of 80 mm and a length of 600 mm, and mixed with filtered air coming from an independent inlet. At the opposite end of the cylinder, the mixed aerosol, still high in water content, should enter an Andersen impactor consisting of six stages through which the incoming aerosol is drawn and separated into different class-sizes (from 7 to 0.65 µm according to the stage). Each stage consists of 400 orifices and a Petri dish, containing an agar culture medium, used as impaction plate. Depending on the diameter of the orifices, droplets of a given class-size impact on the Petri dish and trigger the formation of microbial colonies. The residual aerosol, exiting the impactor lower hose, is safely condensed and filtered before reaching the vacuum pump.

Test runs should be performed with the mask positioned at the entrance of the six-stage impactor, with a test area ≥ 49 cm^2^. The standard method specifies to run the aerosol for 1 min, followed by 1 min of airflow without aerosol generation. Before and after the test runs, a positive control run should be obtained without the mask in place. To check for possible contamination of the apparatus, a negative control run is performed for each experimental session with no mask and a 2 min airflow with no aerosol generation. After the sampling period is completed, the Petri dishes should be removed from the impactor and incubated at 37° ± 2 °C for 24 h. Colony forming units (CFUs) should be enumerated using the “positive hole” correction^30^ for stages 3–6, as described by Andersen^28^. The distribution of the CFU counts in the six stages indicates the distribution of the impacting droplets divided by size classes. The Mean Particle Size (MPS) of the generated aerosol is measured for each testing session without the mask, according to the following equation:

2 $$\mathrm{MPS }= \frac{{\sum }_{i=1}^{6}({P}_{i}x{C}_{i})}{{\sum }_{i=1}^{6}{C}_{i}}$$

where P_i_ is the particle diameter having the 50% probability of being captured by the ith stage of the impactor, and *C*_*i*_ is the number of CFUs counted at the ith stage. The standard specifies the test should be performed using a Mean Particle Size of the mixed aerosol of positive control runs in the range from 2.7 to 3.3 µm. Moreover, the standard method requires that the average number of CFUs in the two positive controls should be between 1700 and 3000 to guarantee both statistical validity of the results and the possibility to correctly enumerate the CFUs on the Petri dishes obtained from the positive control runs.

The BFE is measured indirectly, comparing results of the positive controls to the results of tests when the mask acts as a filter. BFE is expressed as a percentage and computed according to equation below:

3 $$\mathrm{BFE }(\mathrm{\%}) =\frac{{({\sum }_{i=1}^{6}{C}_{i})}_{control}-{({\sum }_{i=1}^{6}{C}_{i})}_{test}}{{({\sum }_{i=1}^{6}{C}_{i})}_{control}}\times 100$$

where the total number of CFUs that pass through the material of the face mask ${({\sum }_{i=1}^{6}{C}_{i})}_{test}$ and the number of CFUs of the incoming aerosol ${({\sum }_{i=1}^{6}{C}_{i})}_{control}$ are considered.

According to the standard method, BFE should be measured on at least five samples and the value of each mask sample should conform requirements specified in clause 5.2.7 of EN 14683:2019^1^.

### Tuning the BFE method using the surrogate microbial strain *Staphylococcus epidermidis* ATCC 12228

*Staphylococcus epidermidis* ATCC 12228, Biohazard group 1, was identified as candidate surrogate of *Staphylococcus aureus* ATCC 6538. The strain was maintained at + 4 °C on Tryptone soy agar, (TSA) (Oxoid CM0131B) and cultivated in Tryptone soy broth (TSB) (Oxoid CM0129) at 37 °C.

A linear calibration curve, correlating the values of optical density at 600 nm (OD_600_) with the microbial concentration (CFU/mL) was obtained in the optical density range between 0.1 and 0.3 (PerkinElmer Lambda 465). To this aim, cultures in liquid medium were obtained by inoculating three colonies, withdrawn from a 24 h-old culture of *S. epidermidis* on TSA, into 30 mL of TSB using a 50 mL centrifuge tube. The tube was incubated, under agitation at 150 rpm, for 24 ± 1 h. At the end of the incubation period, aliquots of the bacterial suspension were diluted with PW (Oxoid CM0509) to obtain OD_600_ values ranging between 0.1 and 0.3. For the CFU count, these bacterial suspensions were further diluted, and 100 µL aliquots were spread on TSA, and incubated at 37 °C for 24 h. Each sample has been prepared in triplicates.

**Figure 4:**
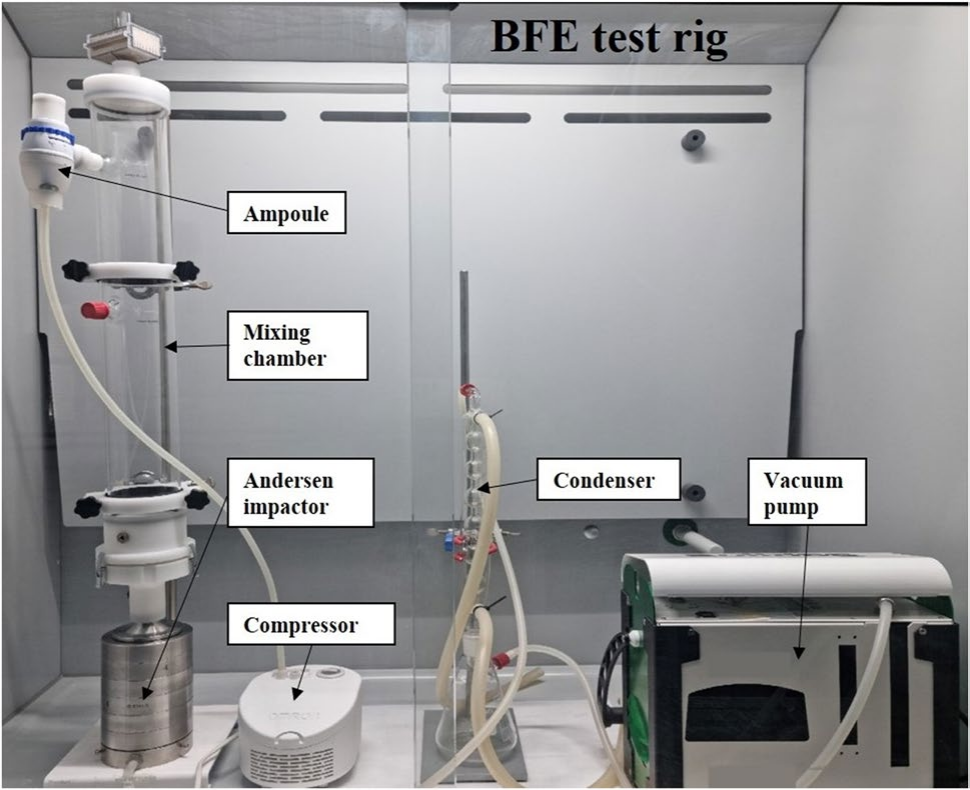
BFE test rig set up used to tune the BFE test method using *S. epidermidis* .

The tuning of the BFE method, using the surrogate microbial strain *S. epidermidis* ATCC 12228, has been performed using the BFE test rig shown in Fig. 4. The BFE apparatus was designed according to the European Standard EN 14683:2019 + AC. The aerosol flow was maintained inside the system by means of a vacuum pump (XEarPro Srl, Cogliate, MB, Italy). The apparatus was equipped with a glass mixing chamber, 60 cm long, with a diameter of 8 cm, and with a 6 stages Andersen impactor, ranging from 7 to 0.65 µm. The aerosol generation was obtained by means of a “Omron C101 Essential” compressor and the bacterial suspension was poured into the “Omron A3 Complete” ampoule with an aerosol nominal mean diameter of 10 µm (Omron Healthcare Europe B.V., Hoofddorp, The Netherlands). The flow rate at the nebulizer was 0.7 ml/min. The test area in the presence of a mask specimen was equal to 49 cm^2^.

The ability of the test rig to generate reproducible aerosol characteristics, in terms of amount and size distribution, was assessed by evaluating the total CFU count and the MPS value in a series of independent positive control runs, without any surgical mask in place. Data were collected in different experimental sessions over one year, considering the influence of variable environmental conditions in term of air relative humidity and temperature. These data allowed us to identify the optimal bacterial concentration (OD_600_) and the required dilutions in PW to obtain a total CFU count and MPS value according to those specified by the European Standard EN 14683:2019 + AC.

Repeatability of BFE test was then conducted on five equivalent samples (same brand, model, lot number, and production date) of a surgical mask available on the market and claimed to be type IIR according to the manufacturer (mask A in Table 4). BFE face mask tests were performed according to the method, specified in Annex B of the European Standard EN 14683:2019 + AC, except that *S. aureus* ATCC 6538 was substituted by the surrogate *S. epidermidis* ATCC 12228 strain. Repeatability standard deviation was calculated according to^24^.

**Table 4:** Characteristics of the mask type tested in the preliminary tuning tests (mask ID: A) and in the comparative BFE measurements (mask ID: B, C, D).

Mask ID	Mask type	Filter material	No. of filter layers	Structure of filter layers	Filter area (cm^2^)	Mask image
A	Surgical type II R	PP	3	S, M, S	195	
B	Community	PP	1	S	361	
C	Surgical type I	PP	3	S, S, S	201	
D	Surgical type II	PP	3	S, M, S	187	

*PP* polypropylene, *S* spun bonded, *M* melt blown.

### Comparative BFE measurements of face masks using *S. aureus and S. epidermidis*

Comparative BFE measurements using both *Staphylococcus aureus* ATCC 6538 and *Staphylococcus epidermidis* ATCC 12228 were performed on three different face mask models. Experiments were performed in a BSL-2 laboratory using the equipment previously described in^12^. Briefly, the test rig included a six-stage Andersen impactor (Tisch six stage viable samples, Tisch Environmental Inc., Village of Cleves, OH, USA) and the same nebulizer (A3 Omron, Osaka, Japan) used in the tuning tests. Bacterial suspensions of both test strains were obtained by the required dilution in PW of an overnight culture in TSB.

Three face mask models (indicated as B, C and D in Table 4), claimed as community, surgical type I, surgical type II masks by the manufacturers, were selected among those available on the market. Ten masks for each model, having the same production date and lot number, were used for the comparative BFE tests. Each mask model was tested in quintuplicate using both the *S. aureus* ATCC 6538 and the *S. epidermidis* ATCC 12228 aerosols. All tests were performed using the same test rig, in a single experimental session to minimize variability due to changes in environmental conditions and operators.

### Data analysis and statistics

In the preliminary tuning test, the microbial concentration of the bacterial suspension (expressed in CFU/mL) was obtained considering the suspension dilution factor and the mean of the triplicate CFU measurements on TSA plates. Correlation between OD values and microbial concentration was finally obtained via linear regression.

In all experiments, CFUs grown on TSA plates from the BFE tests were manually enumerated applying the positive hole correction on plates from impactor stages 3–6. The BFE value of each mask sample was calculated according to Eq. (3). The mean and standard deviation of the BFE values obtained from the five mask samples were finally computed.

For comparative BFE measurements, spectral BFE values were also calculated for the aerodynamic size of each impactor stage, namely 0.65, 1.1, 3.3, 4.7, 7.0 µm using the following equation:

4 $${BFE}_{i}\left(\%\right)=\frac{{({C}_{i})}_{control}-{({C}_{i})}_{test}}{{({C}_{i})}_{control}}\times 100$$

where ${({C}_{i})}_{control}$ and ${({C}_{i})}_{test}$ is the number of CFUs counted at the i^th^ stage of the impactor respectively at control and test runs.

Significant differences in results of BFE test obtained using *S. aureus* and *S. epidermidis* on the three mask models were assessed using ANOVA two-way test followed by Bonferroni post hoc correction*.* The ANOVA analysis was first performed on BFE values as defined by the standard test and then on spectral BFE values corresponding to the aerodynamic size of each impactor stage. Significance was set to p < 0.05. Statistical analysis was performed in Prism 9, GraphPad Software, San Diego, CA, USA.

### Supplementary Information

Supplementary Figures.

## Data Availability

All main data are available in the main text or the supplementary materials. Complementary information upon data used in the analysis are available on reasonable request to corresponding author.
