# Supplementary material for: Staphylococcus epidermidis is a safer surrogate of Staphylococcus aureus in testing bacterial filtration efficiency of face masks
Source: Sci Rep. 2023 Dec 9;13:21807. doi: 10.1038/s41598-023-49005-4 (PMC10710463; doi:10.1038/s41598-023-49005-4)
Supplement: Supplementary file 1 — Supplementary Figures. [file 41598_2023_49005_MOESM1_ESM.docx]

***Staphylococcus epidermidis* is a safer surrogate of *Staphylococcus. aureus* in testing Bacterial Filtration Efficiency of face masks**

Silvia Chiera^1§^, Francesca Bosco^2§*^, Chiara Mollea^2^, Amelia Piscitello^3^, Rajandrea Sethi^3^, Giandomenico Nollo^1^, Iole Caola^4^, Francesco Tessarolo^1^

**SUPPLEMENTARY MATERIAL:**


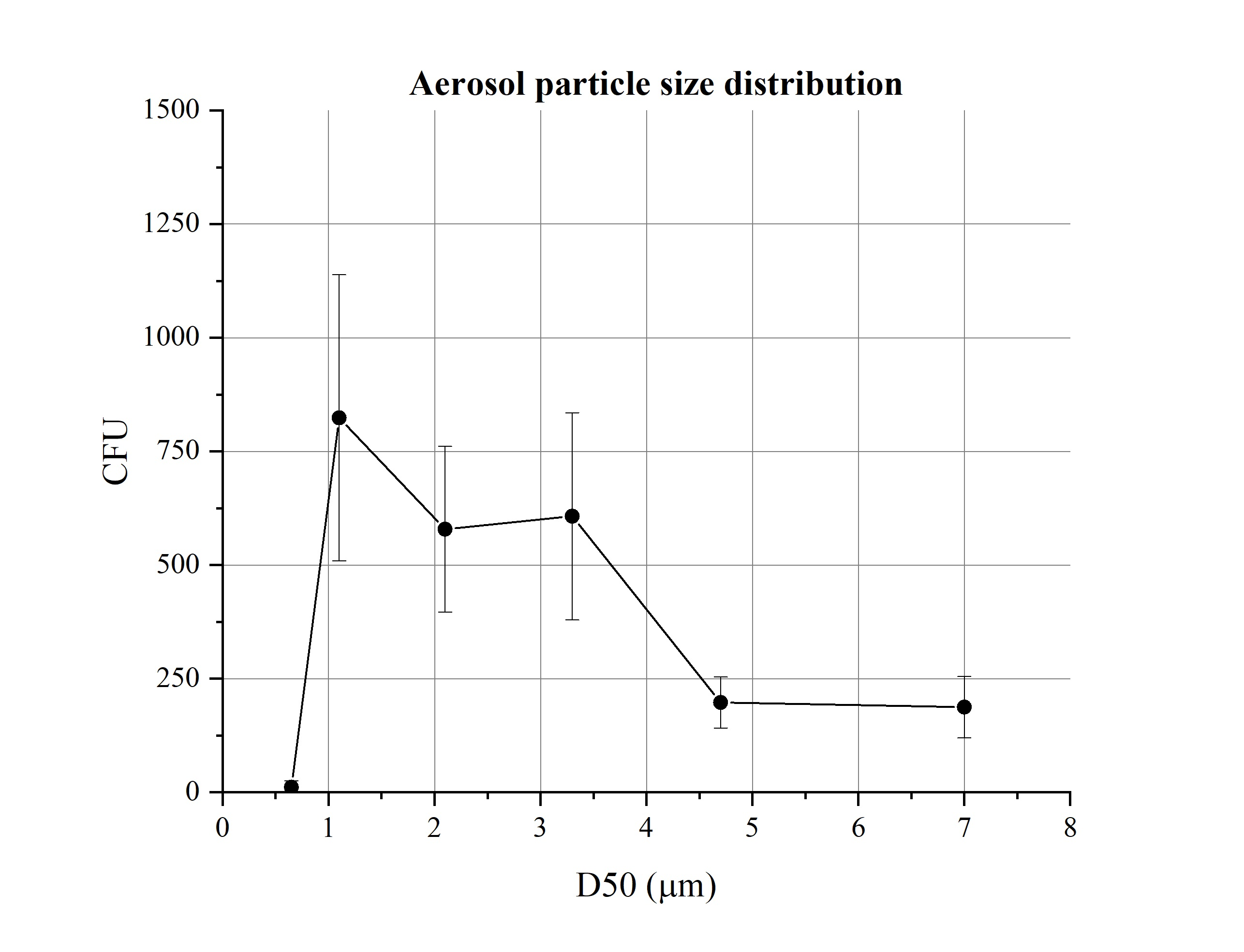


**Supplementary Figure 1:** Aerosol particle size distribution obtained using *S. epidermidis*. Mean values over twelve positive controls, performed in different experimental tests, are presented with their standard deviations.


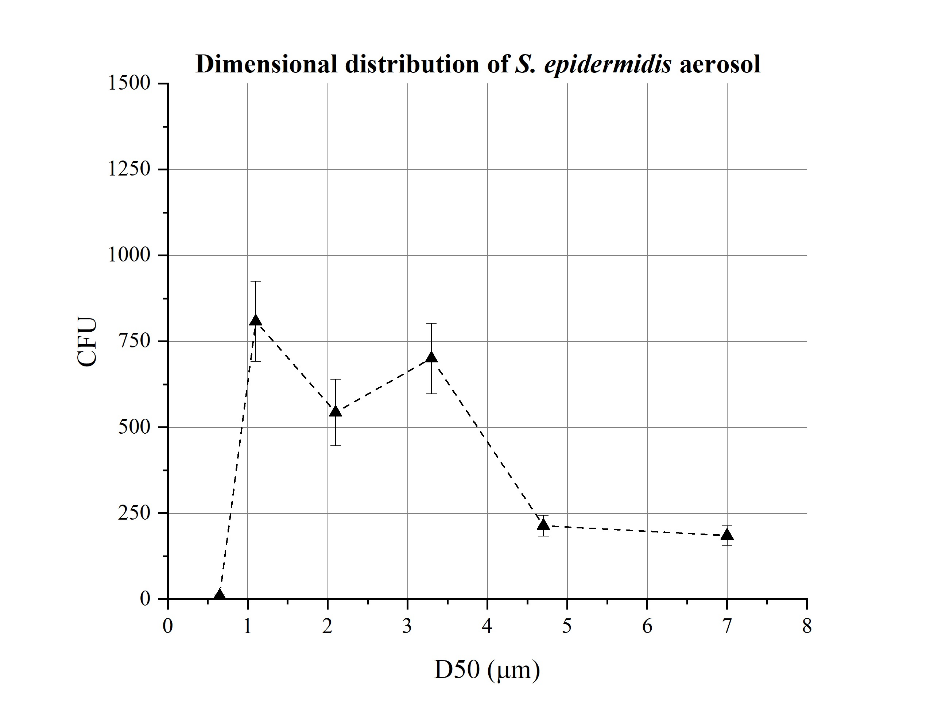


**Supplementary Figure 2:** Graphic representation of the dimensional distribution of the *S. epidermidis* aerosol expressed as mean of the total plate counts of the two positive controls.
